# Long-term dynamics of *Mycoplasma conjunctivae* at the wildlife-livestock interface in the Pyrenees

**DOI:** 10.1371/journal.pone.0186069

**Published:** 2017-10-09

**Authors:** Xavier Fernández-Aguilar, Oscar Cabezón, Joachim Frey, Roser Velarde, Emmanuel Serrano, Andreu Colom-Cadena, Giuseppina Gelormini, Ignasi Marco, Gregorio Mentaberre, Santiago Lavín, Jorge Ramón López-Olvera

**Affiliations:** 1 Servei d’Ecopatologia de Fauna Salvatge (SEFaS), Departament de Medicina i Cirurgia, Universitat Autònoma de Barcelona, Bellaterra, Spain; 2 IRTA, Centre de Recerca en Sanitat Animal (CReSA, IRTA-UAB), Campus de la Universitat Autònoma de Barcelona, Bellaterra, Spain; 3 Institute of Veterinary Bacteriology, Vetsuisse Faculty, University of Bern, Bern, Switzerland; 4 Departamento de Biologia & Cesam, Universidad de Aveiro (UA), Aveiro, Portugal; The University of Melbourne, AUSTRALIA

## Abstract

Functional roles of domestic and wild host populations in infectious keratoconjunctivitis (IKC) epidemiology have been extensively discussed claiming a domestic reservoir for the more susceptible wild hosts, however, based on limited data. With the aim to better assess IKC epidemiology in complex host-pathogen alpine systems, the long-term infectious dynamics and molecular epidemiology of *Mycoplasma conjunctivae* was investigated in all host populations from six study areas in the Pyrenees and one in the Cantabrian Mountains (Northern Spain). Detection of *M*. *conjunctivae* was performed by qPCR on 3600 eye swabs collected during seven years from hunted wild ungulates and sympatric domestic sheep (n = 1800 animals), and cluster analyses of the strains were performed including previous reported local strains. *Mycoplasma conjunctivae* was consistently detected in three Pyrenean chamois (*Rupicapra p*. *pyrenaica*) populations, as well as in sheep flocks (17.0% of sheep) and occasionally in mouflon (*Ovis aries musimon*) from the Pyrenees (22.2% in one year/area); statistically associated with ocular clinical signs only in chamois. Chamois populations showed different infection dynamics with low but steady prevalence (4.9%) and significant yearly fluctuations (0.0%– 40.0%). Persistence of specific *M*. *conjunctivae* strain clusters in wild host populations is demonstrated for six and nine years. Cross-species transmission between chamois and sheep and chamois and mouflon were also sporadically evidenced. Overall, independent *M*. *conjunctivae* sylvatic and domestic cycles occurred at the wildlife-livestock interface in the alpine ecosystems from the Pyrenees with sheep and chamois as the key host species for each cycle, and mouflon as a spill-over host. Host population characteristics and *M*. *conjunctivae* strains resulted in different epidemiological scenarios in chamois, ranging from the fading out of the mycoplasma to the epidemic and endemic long-term persistence. These findings highlight the capacity of *M*. *conjunctivae* to establish diverse interactions and persist in host populations, also with different transmission conditions.

## Introduction

Complex systems involving several hosts suppose a challenge for disease ecology studies [1,2]. In the absence of genetics, patterns of incidence and pathogen prevalence are often used to assess the epidemiological roles of host populations, but they have a number of limitations that may infer wrong functional roles [3]. Even with genetics and the evidence of cross-species transmission, low resolution of spatiotemporal data may not provide a comprehensive insight of the host-pathogen system [4]. Furthermore, the identification of reservoir host populations can be especially critical in pathogens that can establish diverse interactions with its hosts, resulting in different clinical outcomes and epidemiological scenarios [5,6].

An illustrative example are infections by *Mycoplasma conjunctivae* at the wildlife-livestock interface in alpine ecosystems, where there is no clear consensus of the functional roles of wild and domestic hosts [7,8]. *Mycoplasma conjunctivae* is the causative agent of infectious keratoconjunctivitis (IKC), which is a highly contagious ocular disease that severely affects Caprinae [8]. Clinical signs of IKC are associated with ocular damage and inflammation, which causes visual impairment and blindness [9]. Disease is usually transient and spontaneous clinical recovery is a common course of the infection [10,11]. However, clinical signs may progress to staphyloma and perforation of the cornea. Mortality in wild hosts can range locally from 5 to 27% and is derived from blindness of the animals that either starve or die because of traumatic accidents [12,13].

Historical records of IKC in wild mountain ungulates date back to the beginning of the 20^th^ century in the European Alps [14]. Since then, outbreaks of IKC have been documented in wild populations from almost all European mountain ranges [15–17], and is considered one of the main diseases of mountain ungulates [8]. Infectious keratoconjunctivitis outbreaks are locally perceived as problematic because of the visual impressiveness of the disease and the economic impact in hunting revenues, which usually decrease due to hunting restrictions [15]. Several wild hosts have been described suffering from IKC, chamois (*Rupicapra* spp.), Alpine ibex (*Capra ibex*), Iberian ibex (*Capra pyrenaica*), mouflon (*Ovis aries musimon*), the Himalayan tahr (*Hemitragus jemlahicus*) and bighorn sheep (*Ovis canadensis*) [6,8,18]. Among them, chamois are the most common and widespread species in Europe and the one that have suffered more frequent and severe IKC outbreaks [12,15,17,19,20]. Infectious keratoconjunctivitis can therefore exert strong influence on chamois population dynamics [13,15,21]. Domestic Caprinae, i.e. sheep and goats, also undergo spontaneous IKC outbreaks, although in general the health impact is comparatively lower than in their wild counterparts [22,23].

Among Southern chamois (*Rupicapra pyrenaica*), IKC was first described in Pyrenean chamois (*Rupicapra p*. *pyrenaica*) in mid 20^th^ century [24], but it was not until 1980–1981 when the first severe IKC outbreak arose in the Central Pyrenees spreading throughout all the massifs in successive and massive events [14,25]. Conversely, no similar IKC outbreaks but only sporadic ocular clinical signs have been described in the Cantabrian chamois (*Rupicapra p*. *parva*)[24].

In alpine ecosystems, several caprine species that are competent hosts for *M*. *conjunctivae* dwell around meadows [26]. Domestic caprines seasonally (May-November) graze free-range in mountain pastures and interactions among wild and domestic ruminants have been broadly documented [27]. Yet the same *M*. *conjunctivae* strains were found in sheep and chamois suffering from IKC in the same area from the Swiss Alps, indicating cross-transmission between these species [28]. This finding suggested that epidemiological cycles involving wild and domestic hosts may occur in alpine ecosystems. However, the epidemiology of *M*. *conjunctivae* may vary considerably among host species. While *M*. *conjunctivae* mostly occurs endemically and at high prevalence in sheep flocks [22,29], wild host populations most commonly suffer from IKC outbreak events [12,17,30], and *M*. *conjunctivae* have been reported not to persist in chamois populations from Eastern Switzerland [19]. The health significance of *M*. *conjunctivae* infection is also different and a higher proportion of asymptomatic infections occurs in domestic sheep as compared to wild hosts [22,31], which in turn suffer from more severe clinical signs [9,15]. Altogether, these differences suggest domestic sheep as an ideal candidate for being a reservoir host to the more susceptible wild hosts [8], and a key species for the *M*. *conjunctivae* maintenance in the alpine ecosystems. Long-term studies that simultaneously consider all competent hosts are needed to yield a comprehensive epidemiological perspective of *M*. *conjunctivae* cycles and the relative epidemiological roles.

In this study, an integrative approach consisting in the assessment of long-term infection dynamics and molecular subtyping of *M*. *conjunctivae* strains is performed in host-pathogen alpine systems within the Southern chamois (*R*. *pyrenaica*) distribution range in Spain, in order to elucidate the relative functional roles of the ungulate species in IKC epidemiology and specifically assess *M*. *conjunctive* persistence in wild host populations. The clinical outcome of the *M*. *conjunctivae* infection is also evaluated in all the ungulate community.

## Materials and methods

### Study areas

**Fig 1:**
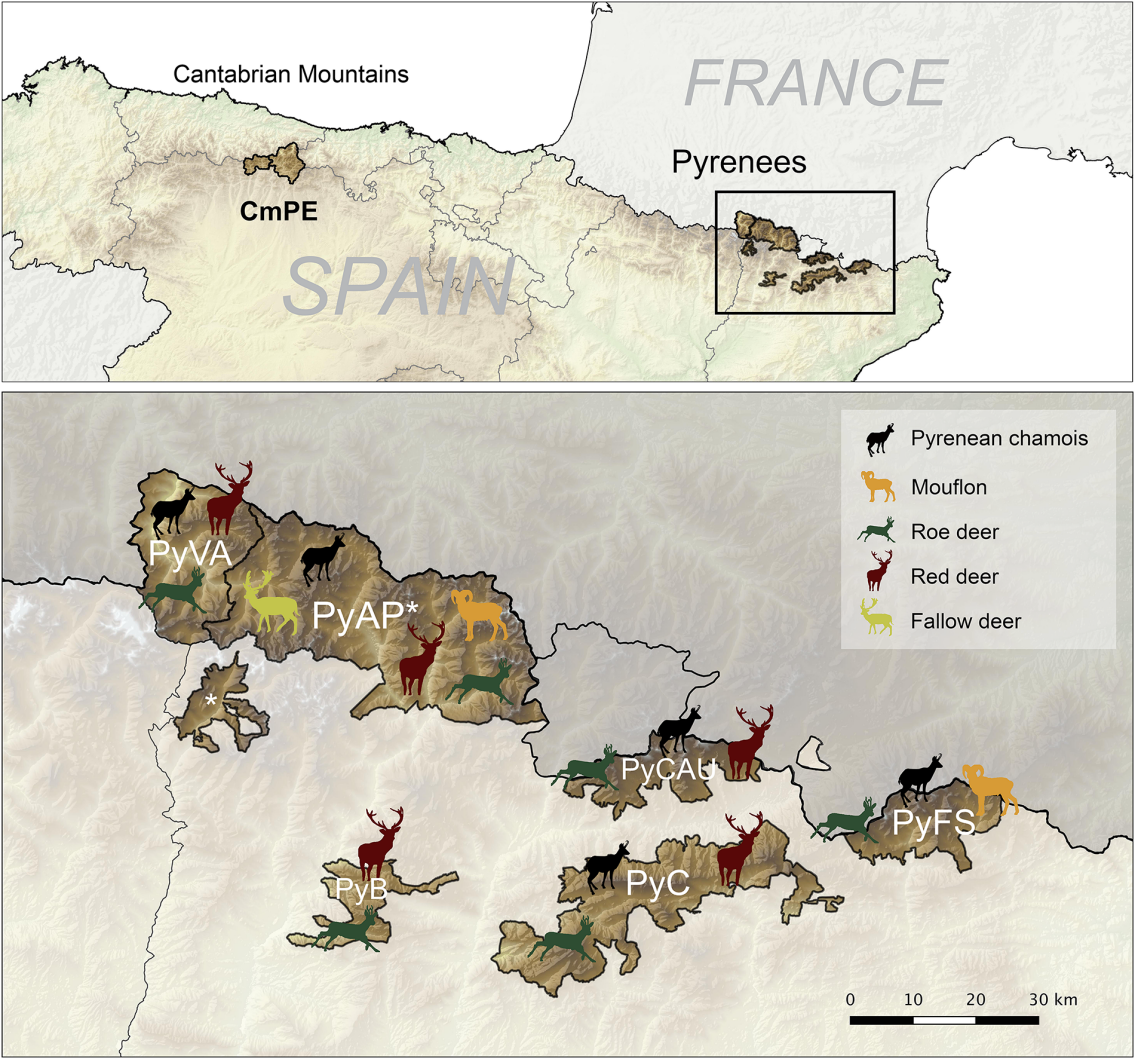
Maps of the study areas and wild ruminant species composition in the Pyrenees. Location of the study areas in Cantabrian Mountains (Picos de Europa—CmPE) and Eastern and Central Spanish Pyrenees. Wild ruminant species composition by study area from the Pyrenees is showed in detail in the image of the bottom: Vall Aran (PyVA), NGR Alt Pallars (PyAP), NGR Boumort (PyB), NGR Cerdanya-Alt Urgell (PyCAU), NGR Cadí (PyC) and NGR Freser-Setcases (PyFS). The asterisk means the same study area. Chamois in PyB is scarce and not representative of the area (not shown in the map).

This study was performed within the distribution area of Pyrenean chamois and Cantabrian chamois in the two main mountain ranges from Northern Spain, the Pyrenees and the Cantabrian Mountains, respectively. Within these ranges, seven different geographical units were considered for the study, six from the Eastern and Central Pyrenees (Catalonia, NE Spain): Freser-Setcases National Game Reserve (PyFS), Cadí National Game Reserve (PyC), Cerdanya-Alt Urgell National Game Reserve (PyCAU), Boumort National Game Reserve (PyB), Alt Pallars National Game Reserve (PyAP) and Vall Aran Game Reserve (PyVA); and one in the Eastern Cantabrian Mountains (León, N Spain), the Natural Protected Area of Picos de Europa (CmPE) (Fig 1). In each study area, ungulate populations are managed independently along with hunting plans.

These areas are high mountain habitats mostly composed of alpine or subalpine ecosystems with strong seasonal influence, with the exception of PyB which has a dryer climate with a higher Mediterranean and continental influence. Altitude ranges approximately from 800 in the bottom of the valleys to 3100 meters high in the Pyrenees and from 1100 meters to 2600 meters high in the Cantabrian Mountains. Chamois is the most abundant ungulate species in most of the study areas except in PyB, where red deer (*Cervus elaphus*) is the predominant wild ruminant (Fig 1). Chamois population size is estimated yearly with linear transects performed by the rangers and is calculated to be about 7,500 in the six study areas from the Pyrenees and 3,800 in the study area from the Cantabrian Mountains. The chamois density varies among study areas as a result of differential pestivirus die-offs in the Pyrenees [32,33]. Mean minimum chamois abundances per square Km during the study period, calculated as estimated chamois population/area, were: PyFS, 14.7; PyC, 3.3; PyCAU, 2.7; PyAP, 1.8; PyVA, 1.8; CmPE 4.0. Other wild ungulates that cohabit with chamois include roe deer (*Capreolus capreolus*), red deer, mouflon, fallow deer (*Dama dama*) and wild boar (*Sus scrofa*), but with a different ruminant community composition among study areas from the Pyrenees, shown in Fig 1. Wild boars are present in all study areas and fallow deer and mouflon are not present in Cantabrian Mountains (CmPE). Domestic ruminants (i.e. cattle, sheep, and goats) and domestic horses also share these habitats with the wild species during the grazing period (May-November) in all of the study areas.

### Sampling method

A long-term cross-sectional sampling design was performed on alpine wild ungulates hunted during the regular hunting seasons from 2009 to 2015 in the Pyrenees (n = 1556) and from 2010 to 2013 in the Cantabrian Mountains (n = 132). Samples were collected from recently hunted ungulates in proportion to the total number of animals hunted in each study area (Table 1). Four sheep flocks from the Pyrenees that graze in the alpine meadows of three of the study areas (PyVA, PyAP and PyFS) were also sampled in 2014 (around 30 sheep per flock; Table 1).

**Table 1:** Distribution of animals sampled by study areas and species.

Study Area	Chamois	Mouflon	Red deer	Roe deer	Fallow deer	Wild boar	Sheep	TOTAL
Vall Aran (PyVA)	125	-	26	25	-	3	30	209
NGR Alt Pallars (PyAP)	122	7	7	22	14	3	59*	234
NGR Boumort (PyB)	-	-	34	-	-	8	-	42
NGR Cerdanya-Alt Urgell (PyCAU)	24	-	3	10	-	2	-	39
NGR Cadí (PyC)	343	-	40	17	-	11	-	411
NGR Freser-Setcases (PyFS)	592	80	-	33	-	1	23	729
Picos de Europa (CmPE)	88	-	25	18	-	1	-	132
**Total**	1294	87	135	125	14	33	112	1800

*Two sheep flocks.

Samples were taken between the third eyelid and the palpebral conjunctiva with sterile cotton swabs without medium from each eye separately and frozen at -20°C within 24 hours from collection. Basic information of the individuals was also registered, including ocular signs, sex, age based on the annual horn segments for chamois and mouflon [34], date and location. Geographic coordinates were also recorded in PyFS from 2012 to 2015. Age was classified in four categories in chamois according to social behavior and aging process, kids (<1 year), yearling (1–2 years), juvenile (2–3 years) and adults (>3 years).

This study accomplish with current guidelines for ethical use of animals in research following the European (2010/63/EU) and Spanish (R.D. 53/2013) legislations. The approval of an ethic committee was not needed since management and sacrifice of animals were not performed for research purposes. Ungulate wild species studied are not endangered, and its abundant populations are managed along hunting plans, regulated by the competent public administrations. Samples were obtained by the rangers from hunted-harvested wild animals during the regular hunting plans from National Game Reserves and Hunting Reserves that belong to public administrations. Both samplings of wild animals and domestic livestock were performed in the frame of health surveillance programs approved by the Departament d′Agricultura, Ramaderia, Pesca, Alimentació i Medi Natural—Generalitat de Catalunya (DARPAMN, the Regional authority in charge of livestock and wildlife management).

### Detection of *Mycoplasma conjunctivae*

Eye swabs were thawed, cut and mixed during one minute with 0.5 mL lysis buffer (100 mM Tris–HCl, pH 8.5, 0.05% Tween 20, 0.24 mg/mL proteinase K) in sterile tubes. The lysates of the cells were obtained by incubating the tubes at 60°C for 60 minutes. Proteinase K was then inactivated at 97°C for 15 minutes [35]. The resulting lysates were directly used as test samples for the molecular detection of *M*. *conjunctivae*.

The presence of *M*. *conjunctivae* DNA in the samples was assessed with a previously described TaqMan real time PCR (qPCR) using primers LPPS-TM-L, LPPS-TM-R, and probe LPPS-TM-FT [35]. Briefly, 2.5 μL of the sample lysates, 900nM of each forward and reverse primer, 300 nM of the probe, 12.5 μL TaqMan^®^2x Universal PCR MasterMix (Applied Biosystems, Warrington, UK) and an exogenous internal positive control (IPC; Applied Biosystems, Warrington, UK) were introduced in each reaction well and nuclease-free water up to a total volume of 25 μL. Cycling conditions were set for 40 cycles at 95°C for 15 s and 60°C for one min, with pre-cycling steps of 50°C for 2 min and 95°C for 10 min. The threshold cycle (Ct) of each sample was defined as the number of cycle at which the fluorescent signal of the reaction crossed the threshold that was set to 0.05.

Samples were analyzed per duplicate and were considered valid only if difference between the replicates was less than one Ct. Samples with Ct≤38 were interpreted as qPCR-positive. All PCR reactions were run on Applied Biosystems^®^ 7500 Fast Real-time PCR system (Applied Biosystems, Warrington, UK).

### *Mycoplasma conjunctivae* subtyping and cluster analyses

The *lppS* gene of *M*. *conjunctivae* encodes for a membrane lipoprotein that is involved in adhesion [36], which variable domain can be used for *M*. *conjunctivae* subtyping and to perform molecular epidemiology analyses [28]. For cluster analyses, samples from this study with Ct values lower than 33 at the qPCR-*M*. *conjunctivae* were considered for sequencing. Available qPCR- *M*. *conjunctivae* positive samples from a previous study that analyzed 439 sheep and goats (19 flocks) from the same study areas and period were also included [22]. To obtain *lppS* gene sequences of these samples, a nested PCR was first performed as described with minor modifications of the primers [28,31] (S1 Table). PCR products were then purified with the High Pure PCR Product Purification Kit (Roche Diagnostics, Rotkreuz, Switzerland). The sequences were determined with the sequencing primers Ser_start2, Ser_start0 and Ser_end0 (S1 Table) using the BigDye termination cycle sequencing kit (Applied Biosystems, Forster City, CA, USA). The resulting sequences were trimmed to contain the region that comprises the nucleotide positions 3935–5035 of the *lppS* gene from *M*. *conjunctivae* type strain HRC/581 (GenBank acc. number AJ318939), which corresponds to the variable *lppS* domain and flanking regions. Alignment and editing of the sequences were performed with the BioEdit software. A phylogenetic analysis of the sequences was then performed by the generation of cluster analyses trees built by the UPGMA statistical method and performing 1000 bootstrap replications [37]. The generation of the phylogenetic tree was performed using MEGA software [38].

For the tree construction, sequences of *M*. *conjunctivae* strains described in previous works from the same study areas were included for comparison (three chamois from PyAP and one mouflon from PyFS) [16], covering a temporal period from 2006 to 2015 (S2 Table). Sequences from other areas (n = 6) and the sequence of the type strain HRC/581 were also included in the tree (S2 Table).

### Data management and statistical analyses

Each individual was considered “infected” if the qPCR was positive in one or both eye swabs. When appropriate, database was organized as recommended for proportion data [39]. *Mycoplasma conjunctivae* apparent prevalence was analyzed to assess 1) the relation between ocular clinical signs and the presence of *M*. *conjunctivae* and 2) the trend of *M*. *conjunctivae* infection probability during the study period in each study area. For the first analyses, a two-sided Chi-squared test for independence was performed. In the second analysis, generalized additive models (GAMs) were fitted with *M*. *conjunctivae* infection as response variable with a binomial distribution and the interaction of year with study area as predictor variables [40]. GAMs can be used to model trends as a nonlinear function of time and provide a framework for testing statistical significance of changes in the response variable frequencies [41]. Known risk factors for *M*. *conjunctivae* infection, such as sex and age category [8], were previously tested with Fisher’s exact tests to be equally represented in all the years for each study area. The absence of residual patterns and other general assumptions were confirmed to validate the model once it was fitted [42]. Statistical significance was set at p<0.05 for all the tests. The interval confidence of apparent prevalences were calculated with the “EpiR” package, the graphics were performed with the “ggplot2” package and the GAMs were implemented in the “mgcv” statistical package, all from the R statistical software [43]. The spatial data representation and mapping was made with the software QGIS 2.14 Essen [44].

## Results

### Prevalence and dynamics of *Mycoplasma conjunctivae* infections

**Fig 2:**
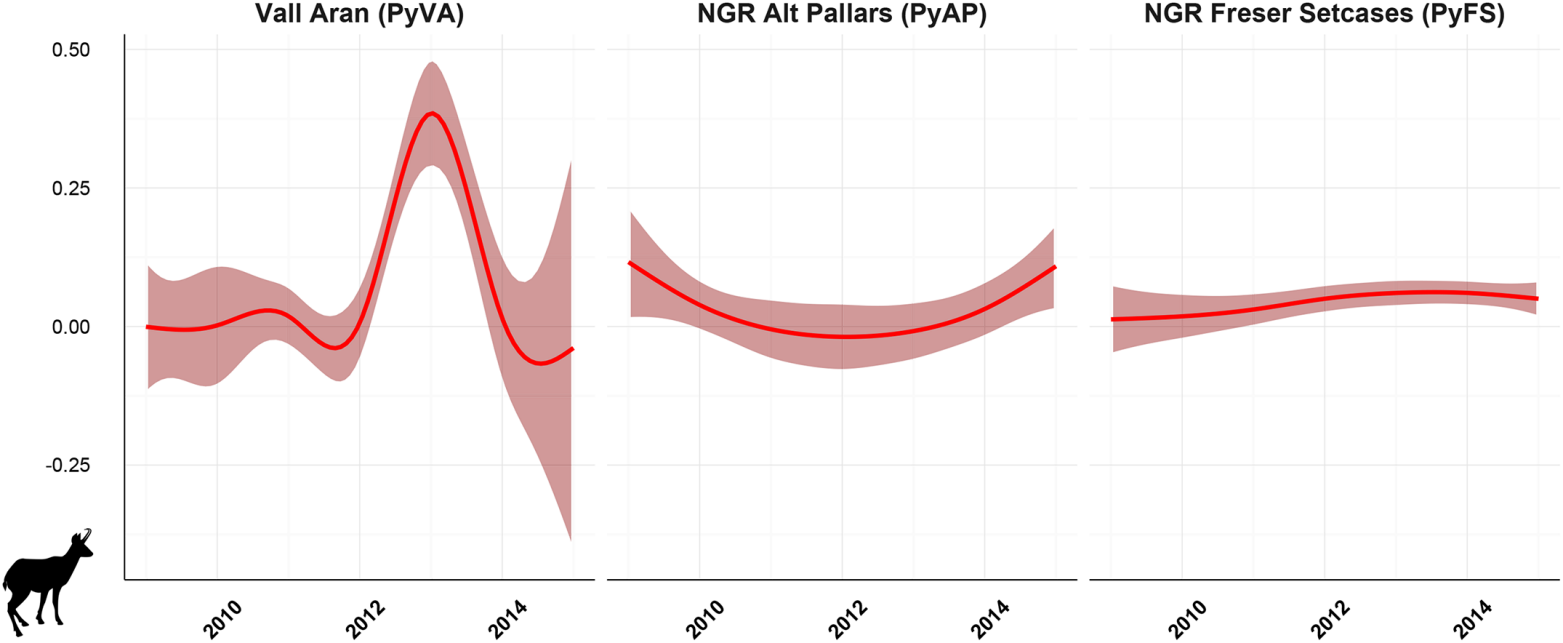
Modeled trend curves of *Mycoplasma conjunctivae* infection in Pyrenean chamois by generalized additive models. Infection indices show evident curve differences by study area, in which the interaction of year with the study area resulted statistically significant in NGR Alt Pallars and Vall Aran, but not in NGR Freser Setcases.

**Fig 3:**
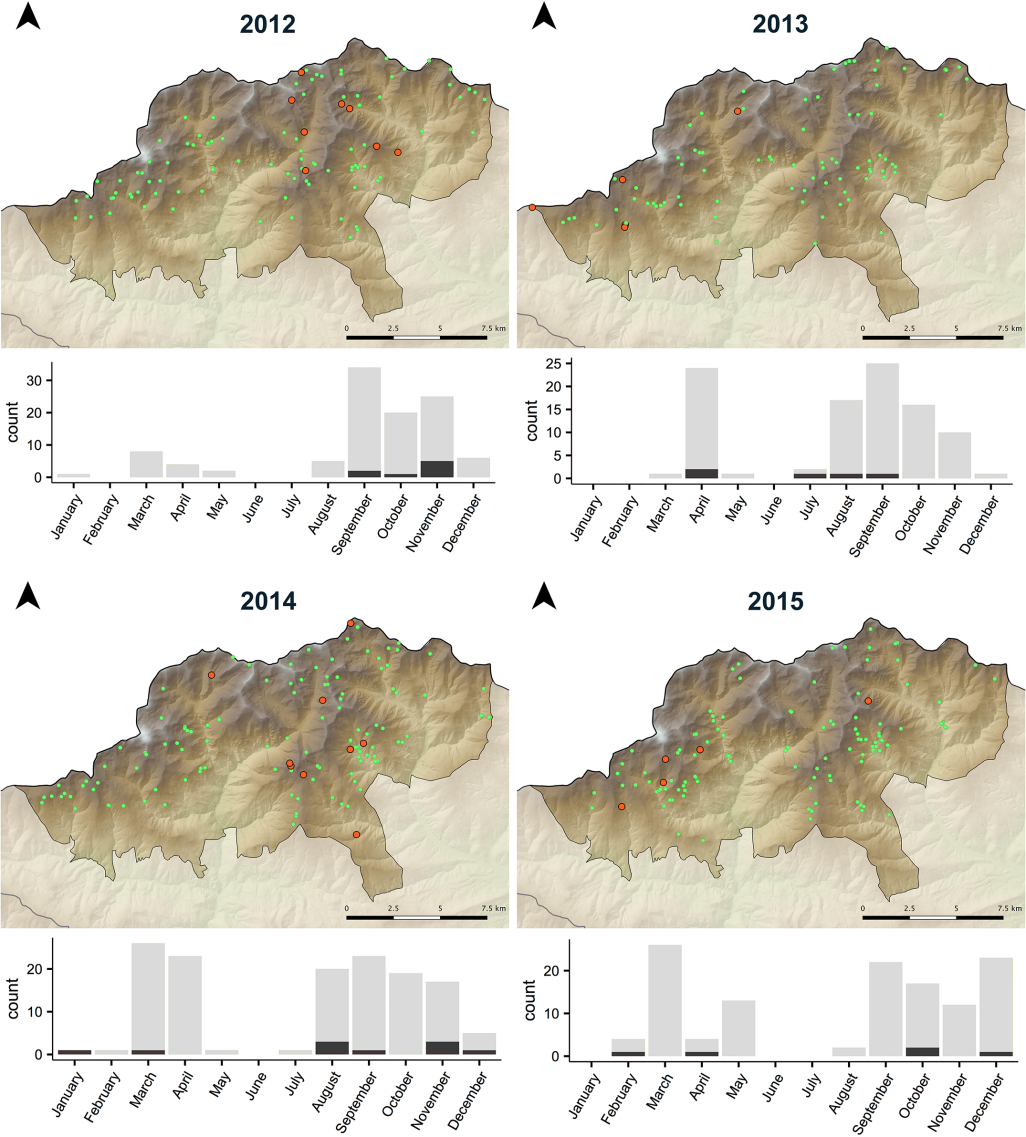
Spatio-temporal distribution of *Mycoplasma conjunctivae* infection in Pyrenean chamois from NGR Freser-Setcasas (PyFS). Orange dots are *M*. *conjunctivae* qPCR-positive chamois and green dots are qPCR-negative chamois. The bar graph at the bottom of each map shows the number of qPCR-positive chamois (dark grey) in total sampled chamois by month that year.

*Mycoplasma conjunctivae* was detected in eye swabs from Pyrenean chamois in three of the study areas (PyFS, PyAP and PyVA). No *M*. *conjunctivae* was detected in Cantabrian chamois (CmPE), as well as in Pyrenean chamois from the rest of study areas from Pyrenees (PyCAU and PyC) (Table 2). Prevalence in Pyrenean chamois population ranged from 2.0% (95% CI 0.1–10.7) to 40.0% (95% CI 19.8–64.2) in years when it was detected (Table 2). The GAM model showed significant differences of *M*. *conjunctivae* infection by year in PyVA (p = 0.025) and PyAP (p<0.001), but not in PyFS (8.12 edf; adjusted R^2^ of 70.4%). Thus, whereas infection indices followed a rather constant trend in PyFS, it changed throughout the study period both in PyAP and more pronouncedly in PyVA, where prevalence peaked in 2013 (Fig 2). No specific *M*. *conjunctivae* distribution pattern was found in Pyrenean chamois from PyFS during 2012–2015 as those described in IKC outbreaks [12,20], and infection cases were found distributed in all seasons and months throughout the reserve (Fig 3). Infection cases detected in PyAP and PyVA were localized in some geographic units within the study areas.

**Table 2:** Temporal trend of *M*. *conjunctivae* prevalence in Pyrenean chamois from three study areas where it was detected.

	2009	2010	2011	2012	2013	2014	2015	Total
**Vall Aran (PyVA)**								
Prev % (Pos/Tot)	0 (0/10)	0 (0/12)	2.0 (1/49)	0 (0/28)	40.0 (6/15)	0 (0/10)	0 (0/1)	5.6 (7/125)
CI 95%	0.0–27.8	0.0–24.2	0.1–10.7	0.0–12.1	19.8–64.2	0.0–27.7	0.0–94.9	2.7–11.1
**NGR Alt Pallars (PyAP)**								
Prev % (Pos/Tot)	20.0 (1/5)	2.5 (1/40)	0 (0/14)	0 (0/6)	0 (0/21)	0 (0/22)	14.3 (2/14)	3.3 (4/122)
CI 95%	1.1–62.4	0.1–12.9	0.0–21.5	0.0–39.0	0.0–15.5	0.0–14.9	4.1–39.9	1.3–8.1
**NGR Freser-Setcases (PyFS)**								
Prev % (Pos/Tot)	3.1 (1/32)	0 (0/8)	0 (0/87)	7.6 (8/105)	5.0 (5/100)	7.3 (10/137)	4.1 (5/123)	4.9 (29/592)
CI 95%	1.8–15.8	0.0–32.4	0.0–4.2	3.9–14.3	2.1–11.2	4.0–12.9	1.7–9.2	3.4–6.9

Prev, prevalence; Pos, positive; Neg, negative; Tot, Total animals analyzed; CI, confidence interval.

Infection of *M*. *conjunctivae* in mouflon was detected only in 2014 in PyFS with a prevalence of 22.2% (95% CI 6.3–54.7). *Mycoplasma conjunctivae* was confirmed in three out of four sheep flocks sampled with an overall prevalence of 17.0% (95% CI 11.1–25.0%) and a within-flock prevalence that ranged from 6.9% (95% CI 1.9–22.0%) to 33.3% (95% CI 19.2–51.2%). *Mycoplasma conjunctivae* was not detected in red deer 0.0% (95% CI 0.0–2.8), roe deer 0.0% (95% CI 0.0–2.9), fallow deer 0.0% (95% CI 0.0–21.5) and wild boar 0.0% (95% CI 0.0–10.4).

### *C*luster analyses of *Mycoplasma conjunctivae* strains

**Fig 4:**
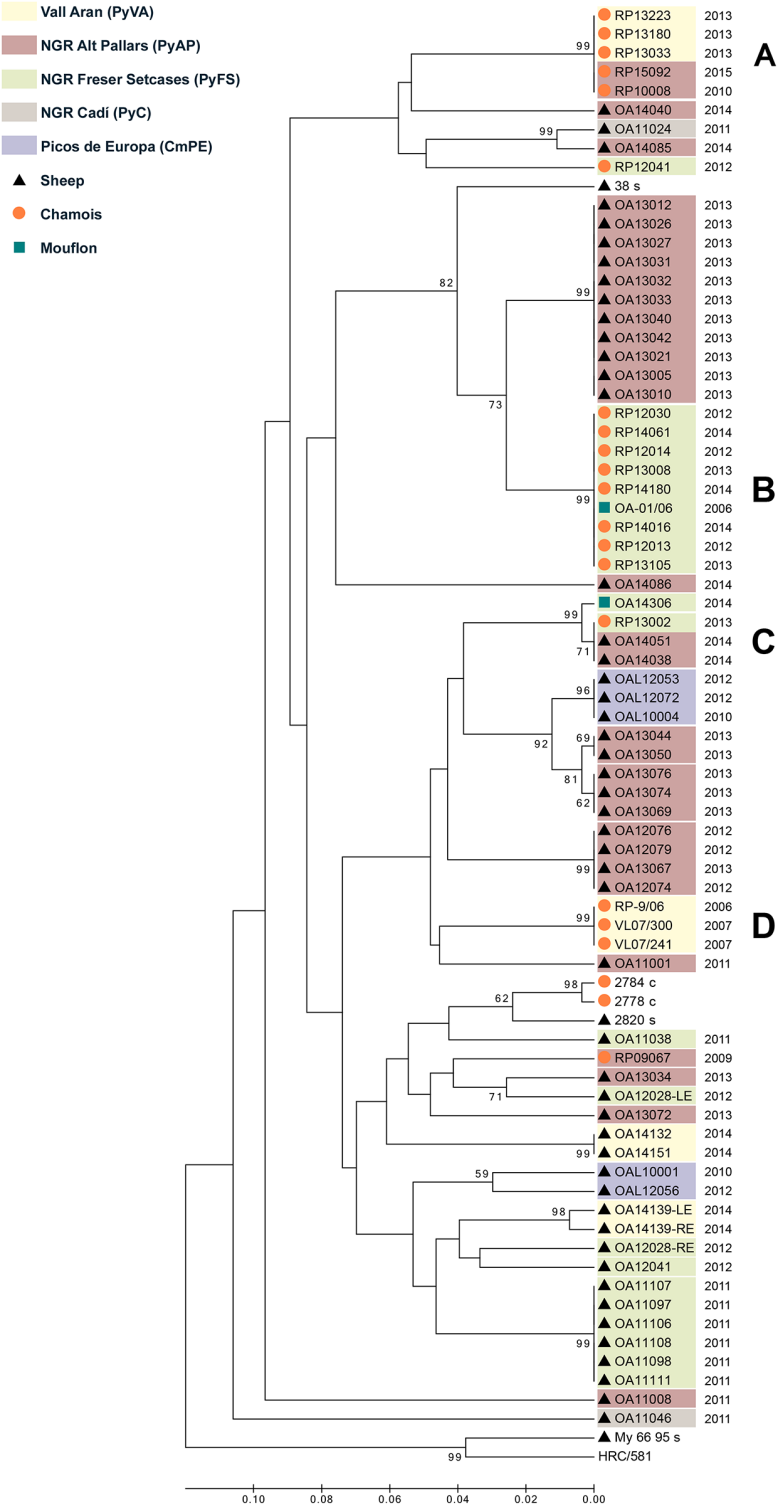
Cluster analyses tree of *Mycoplasma conjunctivae* strains. The tree was inferred using the UPGMA method and including strains identified in chamois, sheep and mouflon during a ten-year period in the Pyrenees and the Cantabrian Mountains. The percentage of replicate trees in which the associated strains clustered together in the bootstrap test (1000 replicates) is shown next to the branches if ≥50%. The four main clusters that include wild ruminants **(A-D)** are shown. Chamois strains mainly clustered by geographic origin in PyFS **(B),** and PyAP together with PyVA as a single epidemiological unit **(A)**. Shared strain clusters among different host species is observed between chamois and mouflon **(B)** and chamois and sheep **(C)**. Sequences from other geographic regions that were included for comparison are showed without background colour. Information associate to each strain is provided in S2 Table.

A total of 81 *M*. *conjunctivae* qPCR-positive samples were sequenced from 65 ruminants, including sheep (8 sheep from 3 flocks included in this study and 40 sheep from 11 flocks in the frame of a previous study [22], grazing in PyFS, PyAP, PyVA, PyC and CmPE), chamois (n = 16; PyFS, PyAP and PyVA) and mouflon (n = 1; PyFS) (S2 Table). In order to simplify the cluster analyses tree, only one *M*. *conjunctivae* strain was considered for the tree construction if the same strain was found in both eyes (n = 67). The tree revealed several clusters of *M*. *conjunctivae* strains, following a host species and geographic pattern (Fig 4). Three main clusters were identified that congregate most of the strains found in wild hosts, one cluster formed by chamois and mouflon strains detected during a nine-year period in PyFS (Fig 4B), another cluster with chamois strains detected during a six-year period in PyAP and PyVA altogether (Fig 4A) and a group clustering chamois strains from PyVA (Fig 4D). Sheep showed higher strain diversity and less clustering by study areas than chamois. Main strain clusters of wild hosts and domestic livestock were neither similar nor related by study area. However, similar strains infecting mouflon and chamois from PyFS (Fig 4B), and sheep and chamois from PyAP and PyFS (Fig 4C), were detected (Fig 4).

### Clinical outcome of *M*. *conjunctivae* infections

Ocular clinical signs were detected in 57/1294 chamois, 3/112 sheep, 1/87 mouflon, 1/125 roe deer, 1/135 red deer, 0/14 fallow deer and 0/33 wild boar, ranging from mild ocular discharge to perforation of the cornea. *Mycoplasma conjunctivae* was significantly (X^2^ = 488.5, df = 1, p<0.001) associated with ocular clinical signs in chamois, but not in the other ungulate species (Table 3). Among the *M*. *conjunctivae* infected chamois, 25.6% (95%CI 14.6–41.1) were asymptomatic, distributed differently among the study areas (PyAP 0/4; PyFS 9/29; PyVA 1/7).

**Table 3:** Detection of *Mycoplasma conjunctivae* by species and ocular clinical signs.

	Total	qPCR Positive	Prevalence % (CI 95%)
**Chamois**			
Clinical signs	57	30	52.6 (39.9–65.0)
No clinical signs	1238	10	0.8 (0.4–1.5)
Total sampled	1294	40	3.1 (2.3–4.2)
**Sheep**			
Clinical signs	3	0	0.0 (0.0–56.1)
No clinical signs	109	19	17.4 (11.4–25.6)
Total sampled	112	19	17.0 (11.1–25.0)
**Mouflon**			
Clinical signs	1	0	0.0 (0.0–94.9)
No clinical signs	86	2	2.3 (0.6–8.1)
Total sampled	87	2	2.3 (0.6–8.0)

Note that clinical signs correspond to any abnormality in the ocular structures or ocular discharge. Species in which *M*. *conjunctivae* was not detected are not shown in the table. CI, Confidence Interval.

## Discussion

### Epidemiological cycles and *M*. *conjunctivae* persistence in host populations

With the aim to better assess the relative functional roles in IKC epidemiology, the long-term infection dynamics and molecular subtyping of *M*. *conjunctivae* was investigated on all potential host species from alpine ecosystems of North Spain. The results further support the specificity of *M*. *conjunctivae* for caprine and ovine hosts [8], being consistently detected in Pyrenean chamois, domestic sheep and occasionally in mouflon. Based on *M*. *conjunctivae* subtyping, the low numbers of related strains shared between competent hosts disclosed domestic and sylvatic cycles, with domestic sheep and Pyrenean chamois as the key host species for each cycle. The overall prevalence of *M*. *conjunctivae* in domestic sheep was relatively high, as it was expected from previous studies performed in the Spanish Pyrenees [15,22] and in the European Alps [29]. Its detection in most of the sheep flocks sampled reinforce sheep as a proper host for the endemic *M*. *conjunctivae* maintenance [23,29]. Specific *M*. *conjunctivae* strain clusters were also recurrently detected in chamois populations in two independent epidemiological areas (PyFS and PyAP-PyVA), indicating that *M*. *conjunctivae* circulated within the sylvatic system at least along the temporal extent of the strains identified (up to nine years).

Long-term detection of *M*. *conjunctivae* clusters has also been demonstrated in wild Caprinae from the Alps, suggesting that *M*. *conjunctivae* persistence may also occur in other alpine systems [7,20]. Contrary to this study, host community of sympatric Caprinae seems to be more important for *M*. *conjunctivae* maintenance in the ecosystems from the Alps, in which a same cluster of strains has been commonly detected in more than one wild host species (i.e. Alpine chamois and ibex)[7,31], as well as both in domestic and wild hosts (i.e. sheep, Alpine chamois and ibex)[28,45]. Population characteristics and allocation/abundance of resources that mainly drive cross-species interactions in natural systems can widely vary between populations and may explain these differences [27,46]. The results of this study indicate that *M*. *conjunctivae* can long-term persist in Pyrenean chamois populations without the significant contribution of other hosts, either domestic or wild, and dismantle the general thought of domestic sheep as the sole reservoir of *M*. *conjunctivae* in the alpine host-pathogen systems. However, *M*. *conjunctivae* might have also faded out in some chamois populations studied (PyCAU and PyC) where historical IKC outbreaks have been documented [14]. Chamois populations from these areas (PyCAU and PyC) have suffered from prior outstanding pestivirus die-offs [32], which may have drop the population under a critical community size for *M*. *conjunctivae* maintenance [47]. The fading out of *M*. *conjunctivae* after an IKC outbreak has been also reported in Alpine chamois from some parts of the European Alps [13,19]. Altogether indicate that population characteristics can drive infection dynamics to result either in long-term persistence or fading out of *M*. *conjunctivae* in Pyrenean chamois, and reinforce similar observations in wild host communities from the Alps (i.e. Alpine chamois and ibex) [19,20]. Previous controversial hypothesis about reservoir hosts inferred by prevalence estimations and molecular epidemiology in a low spatio-temporal frame or studies performed on partial samplings of the host community should be critically revised [3].

The finding of a related strain in chamois and mouflon (PyFS) and in allopatric sheep more than 100 Km away (PyAP) indicates a wide geographic dispersion of this single cluster (Fig 4C). Despite *M*. *conjunctivae* epizootics can cover long distances [15,20], it is unlikely to be caused by a natural spread of *M*. *conjunctivae* under the non-epizootic conditions associated [31]. The limited dispersion movements of chamois [48] suggest that livestock trade and/or long-distance movements of livestock in the Pyrenees may favor the introduction of *M*. *conjunctivae* strains between distant areas [23]. Since both chamois and sheep can maintain *M*. *conjunctivae* as indicated in the present study, cross-transmission between them may have occurred in different past or recent events in the Pyrenees [49], as suggested by the finding of some divergent strains in chamois from each study area (Fig 4). Accordingly, *M*. *conjunctivae* was not detected in Cantabrian chamois, where most of the sympatric sheep flocks were free of *M*. *conjunctivae* [22]. Similar correspondences between the epidemiological scenarios in sympatric sheep and chamois have been also reported for other pathogens in the study area [50,51], highlighting the significance of spillover events among competent hosts at the wildlife-livestock interface, even if its occurrence is rare [52,53]. Even though independent sylvatic cycles accounted for most of the IKC cases in wild hosts from the Pyrenees, the higher prevalence and diversity of *M*. *conjunctivae* strains in sheep suggest that sheep cannot be ruled out as a source of IKC outbreaks in chamois/wild hosts from alpine ecosystems, owing to cross-species transmission of highly virulent strains [28].

### *Mycoplasma conjunctivae* infection dynamics in wild host populations

The different patterns of the *M*. *conjunctivae* infection indices observed in chamois populations (Fig 2) indicates that *M*. *conjunctivae* persistence entailed different infection dynamics including both epidemic and non-epidemic transmissions. Thus, the low but regular *M*. *conjunctivae* detection in PyFS along with the dispersed location of the infection cases agrees with an endemic maintenance of *M*. *conjunctivae* [31,54]. Conversely, the prevalence in PyAP and PyVA fluctuated reaching higher incidence in some years followed by the absence of detection, suggesting a sporadic and localized epizootic spread of *M*. *conjunctivae* [19]. Similar IKC peaks have been observed every three to eight years in chamois populations from neighboring areas in the Eastern and Central Pyrenees [55,56], as well as in the Alps [14,17], but without leading to the high proportion of mortality that occur at the first disease emergence [12]. The dramatic drop of some chamois populations from the Eastern and Central Pyrenees since the pestivirus arose may have also prevented from extensive IKC outbreaks, as occurred in 1981–1983 in these areas [14], or in other parts of the Pyrenees still not affected by the demographic effects of pestivirus [15,21].

Virulence is positively related with mycoplasma transmission in ocular disease [57], which could suggest that the strain clusters that circulate in the two epidemiological areas identified in chamois (PyFS and PyVA-PyAP) differ in virulence. However, population density, which is clearly different in both areas (eight times higher in PyFS), can be also determinant for transmission by influencing connectivity among individuals, groups and subpopulations [47,58]. Since size and fusion rates of chamois groups increase with density [59], a nearly density-dependent transmission may occur if population density is high and groups/subpopulation units are highly connected [60]. The adaptive immune response of hosts does not always prevent mycoplasma re-infection [61–63], which may eventually be enhanced in denser populations and drive mycoplasma-host interaction to an endemic scenario [6], as observed in PyFS. Low population density in group-living ungulates can however shape pathogen transmission to be frequency-dependent if contacts between social groups or subpopulations are not common [60,64]. The scaling of *M*. *conjunctivae* transmission within and between subpopulations may underlay the temporally different disease peaks observed in PyAP and PyVA caused by the same strain cluster (Fig 4)[60,65]. The detection of *M*. *conjunctivae* was not constant in these areas, suggesting that both could be part of a bigger epidemiological unit, probably including part of the French Pyrenees, which would enable the epidemic spread of these strains within a subpopulation and its recurrent detection after a temporal fading out [20].

Despite coexistence and spatial overlap of mouflon with chamois and sheep, and the close interaction that may occur among them [66,67], *M*. *conjunctivae* was detected only sporadically in mouflon. Therefore, mouflon is probably a spill-over host in the systems studied with self-limiting *M*. *conjunctivae* infection in the population.

### Ocular clinical signs and *Mycoplasma conjunctivae*

*Mycoplasma conjunctivae* infections were associated with ocular clinical signs only in chamois and was probably the main etiological cause of ocular disease in the Pyrenean chamois, as previously suggested [16]. These results further indicate a particular susceptibility of chamois to develop ocular disease by *M*. *conjunctivae* infection [7]. Asymptomatic infections in Pyrenean chamois were higher in the endemic *M*. *conjunctivae* area PyFS than when detected associated to epidemics in PyVA and PyAP, which may indicate a higher tolerance to the infection associated to the endemic maintenance. However, high number of asymptomatic *M*. *conjunctivae* infections have been reported in both epizootics and situations with low but regular incidence of IKC cases [6,31]. Clinical signs compatible with IKC were not registered in Cantabrian chamois, which agrees with the absence of *M*. *conjunctivae* detection in this area. Previous reports of keratoconjunctivitis in Cantabrian chamois have been described in the absence of disease outbreaks, but they could not be attributed to *M*. *conjunctivae* nor an infectious origin was ascertained [24].

Although sheep and mouflon may develop IKC due to *M*. *conjunctivae* infection [16,68,69], both species showed a high rate of asymptomatic infections in this study, agreeing with the lack of statistical association between IKC and *M*. *conjunctivae* detection [70]. Altogether highlight differences of susceptibility to the circulating *M*. *conjunctivae* strains among hosts [7,20]. Other infectious agents may also have sporadically caused eye disease in negative qPCR-*M*. *conjunctivae* [70,71], although IKC healing stages in which *M*. *conjunctivae* is not present anymore but evident lesions are still observed probably accounted for most of the Caprinae cases [6].

## Conclusions

Independent *M*. *conjunctivae* sylvatic and domestic cycles mainly occurred at the wildlife-livestock interface in alpine ecosystems from the Pyrenees, indicating that *M*. *conjunctivae* was maintained in some chamois populations without the substantial contribution of other hosts. Furthermore, host population characteristics and *M*. *conjunctivae* strains resulted in different epidemiological scenarios in chamois, ranging from the fading out of the mycoplasma to the epidemic and endemic long-term persistence. Altogether, these findings highlight the capacity of *M*. *conjunctivae* to establish diverse interactions and persist in host populations, also with different transmission conditions. Population characteristics can therefore shape host-mycoplasma interaction and ultimately its functionality in the system. Host transitions of *M*. *conjunctivae* clusters in the alpine ecosystems were also occasionally observed, which indicates that cross-species transmission can be a source of IKC outbreaks.

## Supporting information

S1 Table Primers and probes used in this study for *Mycoplasma conjunctivae* detection and Sanger DNA sequence analysis. (DOCX)

S2 Table Information of the *M*. *conjunctivae* strains included in the cluser analyses. Sequences belonged to strains found in Pyrenean chamois (2006–2007; 2009–2015), mouflon (2006 and 2014) and sympatric sheep (2011–2014) from the Pyrenees and the Cantabrian Mountains. Ocular clinical signs associated to the strain are registered in the “IKC” column. Shaded rows are strains from other areas included for comparison.
(DOCX)
