# Supplementary material for: Long-term dynamics of Mycoplasma conjunctivae at the wildlife-livestock interface in the Pyrenees
Source: PLoS One. 2017 Oct 9;12(10):e0186069. doi: 10.1371/journal.pone.0186069 (PMC5633175; doi:10.1371/journal.pone.0186069)
Supplement: S1 Table — (DOCX) [file pone.0186069.s001.docx]

| **Primers and probes** | **Sequences (5’-3’)** | **Use** |
| --- | --- | --- |
| LPPS-TM-L | CAGCTGGTGTAGCACTTTTTGC | qPCR |
| LPPS-TM-R | TTAACACCTATGCTCTCGTCTTTGA | qPCR |
| LPPS-TM-FT | TGCTTCGACTACCAAATATGATGGTGATCCTCT^a^ | qPCR - probe |
| Serstart3 | TTTAGTAGACTCCACTTCACC | PCR |
| Serstart2 | CACTATACTTAACAGATAGTCC | Nested PCR, Sequencing |
| Serstart0 | ATACTCAAAGTGGAAATAATGGAA | Sequencing |
| Serend0 | GCAACAACAATAGTAAGAGCAG | Sequencing |
| lppTA2 | TTTGATCTCTCCACCTTCAGC | PCR |
| lppTA | GGCACTAATAGTGCGTAATTC | Nested PCR |

^a^5’ 6FAM reporter dye and 3’ TAMRA quencher.
