# Supplementary material for: Long-term dynamics of Mycoplasma conjunctivae at the wildlife-livestock interface in the Pyrenees
Source: PLoS One. 2017 Oct 9;12(10):e0186069. doi: 10.1371/journal.pone.0186069 (PMC5633175; doi:10.1371/journal.pone.0186069)
Supplement: S2 Table — Sequences belonged to strains found in Pyrenean chamois (2006–2007; 2009–2015), mouflon (2006 and 2014) and sympatric sheep (2011–2014) from the Pyrenees and the Cantabrian Mountains. Ocular clinical signs associated to the strain are registered in the “IKC” column. Shaded rows are strains from other areas included for comparison. (DOCX) [file pone.0186069.s002.docx]

| **ID sequence** | **Species** | **Zone** | **Year** | **IKC** | **Herd status** | **GenBank** | **Reference** |
| --- | --- | --- | --- | --- | --- | --- | --- |
| RP-9/06 | Southern chamois | Vall Aran (PyVA) | 2006 | Yes | IKC outbreak | LT708373 | Marco et al., 2009 |
| VL07/241 | Southern chamois | Vall Aran (PyVA) | 2007 | Yes | IKC outbreak | LT708374 | Marco et al., 2009 |
| VL07/300 | Southern chamois | Vall Aran (PyVA) | 2007 | Yes | IKC outbreak | LT708375 | Marco et al., 2009 |
| RP09067 | Southern chamois | NGR Alt Pallars (PyAP) | 2009 | Yes | IKC outbreak | LT708305 | This study |
| RP10008 | Southern chamois | NGR Alt Pallars (PyAP) | 2010 | Yes | IKC outbreak | LT708306 | This study |
| RP12013 | Southern chamois | NGR Freser-Setcases (PyFS) | 2012 | Yes | Sporadic IKC cases | LT708307 | This study |
| RP12014 | Southern chamois | NGR Freser-Setcases (PyFS) | 2012 | Yes | Sporadic IKC cases | LT708308 | This study |
| RP12030 | Southern chamois | NGR Freser-Setcases (PyFS) | 2012 | Yes | Sporadic IKC cases | LT708309 | This study |
| RP12041 | Southern chamois | NGR Freser-Setcases (PyFS) | 2012 | Yes | Sporadic IKC cases | LT708310 | This study |
| RP13002 | Southern chamois | NGR Freser-Setcases (PyFS) | 2013 | Yes | Sporadic IKC cases | LT708311 | This study |
| RP13008 | Southern chamois | NGR Freser-Setcases (PyFS) | 2013 | Yes | Sporadic IKC cases | LT708312 | This study |
| RP13033 | Southern chamois | Vall Aran (PyVA) | 2013 | Yes | IKC outbreak | LT708313 | This study |
| RP13105 | Southern chamois | NGR Freser-Setcases (PyFS) | 2013 | Yes | Sporadic IKC cases | LT708314 | This study |
| RP13180 | Southern chamois | Vall Aran (PyVA) | 2013 | Yes | IKC outbreak | LT708315 | This study |
| RP13223 | Southern chamois | Vall Aran (PyVA) | 2013 | Yes | IKC outbreak | LT708316 | This study |
| RP14016 | Southern chamois | NGR Freser-Setcases (PyFS) | 2014 | Yes | Sporadic IKC cases | LT708317 | This study |
| RP14061 | Southern chamois | NGR Freser-Setcases (PyFS) | 2014 | Yes | Sporadic IKC cases | LT708318 | This study |
| RP14180 | Southern chamois | NGR Freser-Setcases (PyFS) | 2014 | Yes | Sporadic IKC cases | LT708319 | This study |
| RP15092 | Southern chamois | NGR Alt Pallars (PyAP) | 2015 | Yes | IKC outbreak | LT708321 | This study |
| OA-01/06 | Mouflon | NGR Freser-Setcases (PyFS) | 2006 | Yes | No IKC | LT708372 | Marco et al., 2009 |
| OA14306 | Mouflon | NGR Freser-Setcases (PyFS) | 2014 | No | No IKC | LT708320 | This study |
| OAL10001 | Sheep | Picos de Europa (CmPE) | 2010 | No | Sporadic IKC cases | LT708322 | This study |
| OAL10004 | Sheep | Picos de Europa (CmPE) | 2010 | No | Sporadic IKC cases | LT708323 | This study |
| OA11001 | Sheep | NGR Alt Pallars (PyAP) | 2011 | No | Sporadic IKC cases | LT708324 | This study |
| OA11008 | Sheep | NGR Alt Pallars (PyAP) | 2011 | No | Sporadic IKC cases | LT708325 | This study |
| OA11024 | Sheep | NGR Cadí (PyC) | 2011 | No | Sporadic IKC cases | LT708326 | This study |
| OA11046 | Sheep | NGR Cadí (PyC) | 2011 | No | Sporadic IKC cases | LT708327 | This study |
| OA11097 | Sheep | NGR Freser-Setcases (PyFS) | 2011 | No | Sporadic IKC cases | LT708328 | This study |
| OA11098 | Sheep | NGR Freser-Setcases (PyFS) | 2011 | No | Sporadic IKC cases | LT708329 | This study |
| OA11106 | Sheep | NGR Freser-Setcases (PyFS) | 2011 | No | Sporadic IKC cases | LT708330 | This study |
| OA11107 | Sheep | NGR Freser-Setcases (PyFS) | 2011 | Yes | Sporadic IKC cases | LT708331 | This study |
| OA11108 | Sheep | NGR Freser-Setcases (PyFS) | 2011 | No | Sporadic IKC cases | LT708332 | This study |
| OA11111 | Sheep | NGR Freser-Setcases (PyFS) | 2011 | No | Sporadic IKC cases | LT708333 | This study |
| OAL12053 | Sheep | Picos de Europa (CmPE) | 2012 | No | Sporadic IKC cases | LT708334 | This study |
| OAL12056 | Sheep | Picos de Europa (CmPE) | 2012 | No | Sporadic IKC cases | LT708335 | This study |
| OAL12072 | Sheep | Picos de Europa (CmPE) | 2012 | No | Sporadic IKC cases | LT708336 | This study |
| OA12028-LE* | Sheep | NGR Freser-Setcases (PyFS) | 2012 | No | Sporadic IKC cases | LT708337 | This study |
| OA12028-RE* | Sheep | NGR Freser-Setcases (PyFS) | 2012 | No | Sporadic IKC cases | LT708338 | This study |
| OA12038 | Sheep | NGR Freser-Setcases (PyFS) | 2012 | No | Sporadic IKC cases | LT708339 | This study |
| OA12041 | Sheep | NGR Freser-Setcases (PyFS) | 2012 | No | Sporadic IKC cases | LT708340 | This study |
| OA12074 | Sheep | NGR Alt Pallars (PyAP) | 2012 | No | Sporadic IKC cases | LT708341 | This study |
| OA12076 | Sheep | NGR Alt Pallars (PyAP) | 2012 | No | Sporadic IKC cases | LT708342 | This study |
| OA12079 | Sheep | NGR Alt Pallars (PyAP) | 2012 | No | Sporadic IKC cases | LT708343 | This study |
| OA13005 | Sheep | NGR Alt Pallars (PyAP) | 2013 | No | Sporadic IKC cases | LT708344 | This study |
| OA13010 | Sheep | NGR Alt Pallars (PyAP) | 2013 | No | Sporadic IKC cases | LT708345 | This study |
| OA13012 | Sheep | NGR Alt Pallars (PyAP) | 2013 | No | Sporadic IKC cases | LT708346 | This study |
| OA13021 | Sheep | NGR Alt Pallars (PyAP) | 2013 | No | Sporadic IKC cases | LT708347 | This study |
| OA13026 | Sheep | NGR Alt Pallars (PyAP) | 2013 | No | Sporadic IKC cases | LT708348 | This study |
| OA13027 | Sheep | NGR Alt Pallars (PyAP) | 2013 | No | Sporadic IKC cases | LT708349 | This study |
| OA13031 | Sheep | NGR Alt Pallars (PyAP) | 2013 | No | Sporadic IKC cases | LT708350 | This study |
| OA13032 | Sheep | NGR Alt Pallars (PyAP) | 2013 | No | Sporadic IKC cases | LT708351 | This study |
| OA13033 | Sheep | NGR Alt Pallars (PyAP) | 2013 | No | Sporadic IKC cases | LT708352 | This study |
| OA13034 | Sheep | NGR Alt Pallars (PyAP) | 2013 | No | Sporadic IKC cases | LT708353 | This study |
| OA13040 | Sheep | NGR Alt Pallars (PyAP) | 2013 | No | Sporadic IKC cases | LT708354 | This study |
| OA13042 | Sheep | NGR Alt Pallars (PyAP) | 2013 | Yes | Sporadic IKC cases | LT708355 | This study |
| OA13044 | Sheep | NGR Alt Pallars (PyAP) | 2013 | No | Sporadic IKC cases | LT708356 | This study |
| OA13050 | Sheep | NGR Alt Pallars (PyAP) | 2013 | No | Sporadic IKC cases | LT708357 | This study |
| OA13067 | Sheep | NGR Alt Pallars (PyAP) | 2013 | No | Sporadic IKC cases | LT708358 | This study |
| OA13069 | Sheep | NGR Alt Pallars (PyAP) | 2013 | Yes | Sporadic IKC cases | LT708359 | This study |
| OA13072 | Sheep | NGR Alt Pallars (PyAP) | 2013 | No | Sporadic IKC cases | LT708360 | This study |
| OA13074 | Sheep | NGR Alt Pallars (PyAP) | 2013 | No | Sporadic IKC cases | LT708361 | This study |
| OA13076 | Sheep | NGR Alt Pallars (PyAP) | 2013 | No | Sporadic IKC cases | LT708362 | This study |
| OA14038 | Sheep | NGR Alt Pallars (PyAP) | 2014 | No | Sporadic IKC cases | LT708363 | This study |
| OA14040 | Sheep | NGR Alt Pallars (PyAP) | 2014 | No | Sporadic IKC cases | LT708364 | This study |
| OA14051 | Sheep | NGR Alt Pallars (PyAP) | 2014 | No | Sporadic IKC cases | LT708365 | This study |
| OA14085 | Sheep | NGR Alt Pallars (PyAP) | 2014 | No | Sporadic IKC cases | LT708366 | This study |
| OA14086 | Sheep | NGR Alt Pallars (PyAP) | 2014 | No | Sporadic IKC cases | LT708367 | This study |
| OA14132 | Sheep | Vall Aran (PyVA) | 2014 | No | Sporadic IKC cases | LT708368 | This study |
| OA14139-LE* | Sheep | Vall Aran (PyVA) | 2014 | No | Sporadic IKC cases | LT708369 | This study |
| OA14139-RE* | Sheep | Vall Aran (PyVA) | 2014 | No | Sporadic IKC cases | LT708370 | This study |
| OA14151 | Sheep | Vall Aran (PyVA) | 2014 | No | Sporadic IKC cases | LT708371 | This study |
| 38 s | Sheep | Swiss Alps | 2000 | Yes | Unknown | LT708379 | Belloy et al., 2003 |
| 2820 s | Sheep | Swiss Alps | 2001 | Yes | Unknown | LT708377 | Belloy et al., 2003 |
| 2778 c | Alpine chamois | Austrian Alps | 2000 | Yes | Unknown | LT708376 | Belloy et al., 2003 |
| 2784 c | Alpine chamois | Austrian Alps | 2000 | Yes | Unknown | LT708380 | Belloy et al., 2003 |
| My 66 95 s | Sheep | Croatia^a^ | 1995 | Yes | Unknown | LT708378 | Giacometti et al., 1999 |
| HRC/581 | Type strain – Sheep | United States of America | 1972 | Yes | Unknown | LT708381 | Barile et al., 1972 |
| *Same animal with diferent strain in each eye.  ^a^ Isolate from sheep that was imported from Australia | | | | | | | |
